# In Vivo Stability of Electronic Intraocular Lens Implant for Corneal Blindness

**DOI:** 10.1167/tvst.14.6.33

**Published:** 2025-06-26

**Authors:** Ibraim V. Vieira, Victoria H. Fan, Michael W. Wiemer, Brian E. Lemoff, Kishan S. Sood, Maria Julia Mussa, Charles Q. Yu

**Affiliations:** 1 Byers Eye Institute, Stanford University School of Medicine, Palo Alto, CA, USA; 2 Department of Ophthalmology, Universidade Federal de São Paulo, São Paulo, Brazil; 3 Mojo Vision, Saratoga, CA, USA

**Keywords:** keratoprosthesis, microdisplay, corneal blindness, intraocular lens, projection

## Abstract

**Purpose:**

Electronic technology can add new function to intraocular lenses, including the treatment of corneal blindness. However, it is not known if such an implant can be stably implanted within a living eye over time. This study investigates the long-term stability and safety of an intraocular lens-shaped implant with an embedded electronic microdisplay for potential use in treating corneal blindness.

**Methods:**

Five intraocular implants containing a nonfunctional microdisplay and projection optic were surgically implanted into five rabbits after removal of their crystalline lenses. This blocks the natural pathway of light into the eye. The rabbits were monitored over 6 months with photography and biometry to assess the centration and axial stability of the implants.

**Results:**

All implants were successfully implanted and remained stable over the 6-month trial. The average distance from the cornea center was 0.868 ± 0.442 mm at 1 month and 0.851 ± 0.591 mm at 6 months. Anterior chamber depth, representing axial stability, was 4.362 ± 0.213 mm at 1 month and 4.351 ± 0.218 mm at 6 months. While posterior capsular opacification and iris adhesions were observed, no major complications occurred.

**Conclusions:**

This study is the first to demonstrate long-term stability of an intraocular lens-shaped implant containing an electronic display and optical system. These findings suggest that such implants are viable and safe, supporting their potential as a treatment for corneal blindness and other broader applications.

**Translational Relevance:**

Evidence of safety and stability of electronic intraocular lenses in animals paves the way for the study of this emerging field of medical implants in humans.

## Introduction

Cornea disease or dysfunction can result in loss of clarity, which results in blindness due to the inability of visual imagery to enter the eye. The treatment for cornea opacity is corneal transplantation, but this relies on donor tissue availability. A total of 12.7 million patients are on waiting lists for cornea transplants worldwide.^1^ The procedure also carries risks, including immunologic rejection, graft failure, and the need for lifelong monitoring. Some clinical cases, such as chemical burns, vascularized corneas, or a history of transplant rejection, may benefit from the use of artificial corneal prostheses, known as keratoprostheses. Keratoprostheses are nonbiologic transparent implants placed through the cornea and used to treat cases where transplants are not available or are expected to fail.^2^^–^^6^ They have been successful in restoring vision in some patients but are limited by a high rate of complications such as inflammatory membranes and optic neuropathy.^7^

This study explores the potential of solving the problem of corneal opacity by replacing the crystalline lens with an electronic intraocular lens. The weight of the crystalline lens in air is ∼225 mg in adults and less when submerged in the aqueous environment of the eye.^8^ It is removed from the eye when it develops into a cataract and replaced with an intraocular lens (IOL) implant.^9^ Over four million intraocular lenses are implanted a year in the United States in cataract surgeries. Most intraocular lenses are simple discs of acrylic 6 to 7 mm in diameter, less than 1 mm in thickness, with arms (haptics), and weighing less than 20 mg in air. There are bigger IOL-like devices that seek to magnify vision for patients with central vision loss due to macular degeneration. These telescopic intraocular lenses or intraocular telescopes include the IOL VIP (Seleko, Pontecorvo, Italy), LMI mirror implant (Optolight Vision Technologies, Herzlia, Israel), IOL AMD (London Eye Hospital Pharma, London, UK), Orilens (Optolight Vision Technologies, Herzlia, Israel), Scharioth Macular lens (Medicontur Medical Engineering, Zsámbék, Hungary), and Centrasight (VisionCare Ophthalmic Technologies, Saratoga, CA, USA).^10^

**Figure 1.**
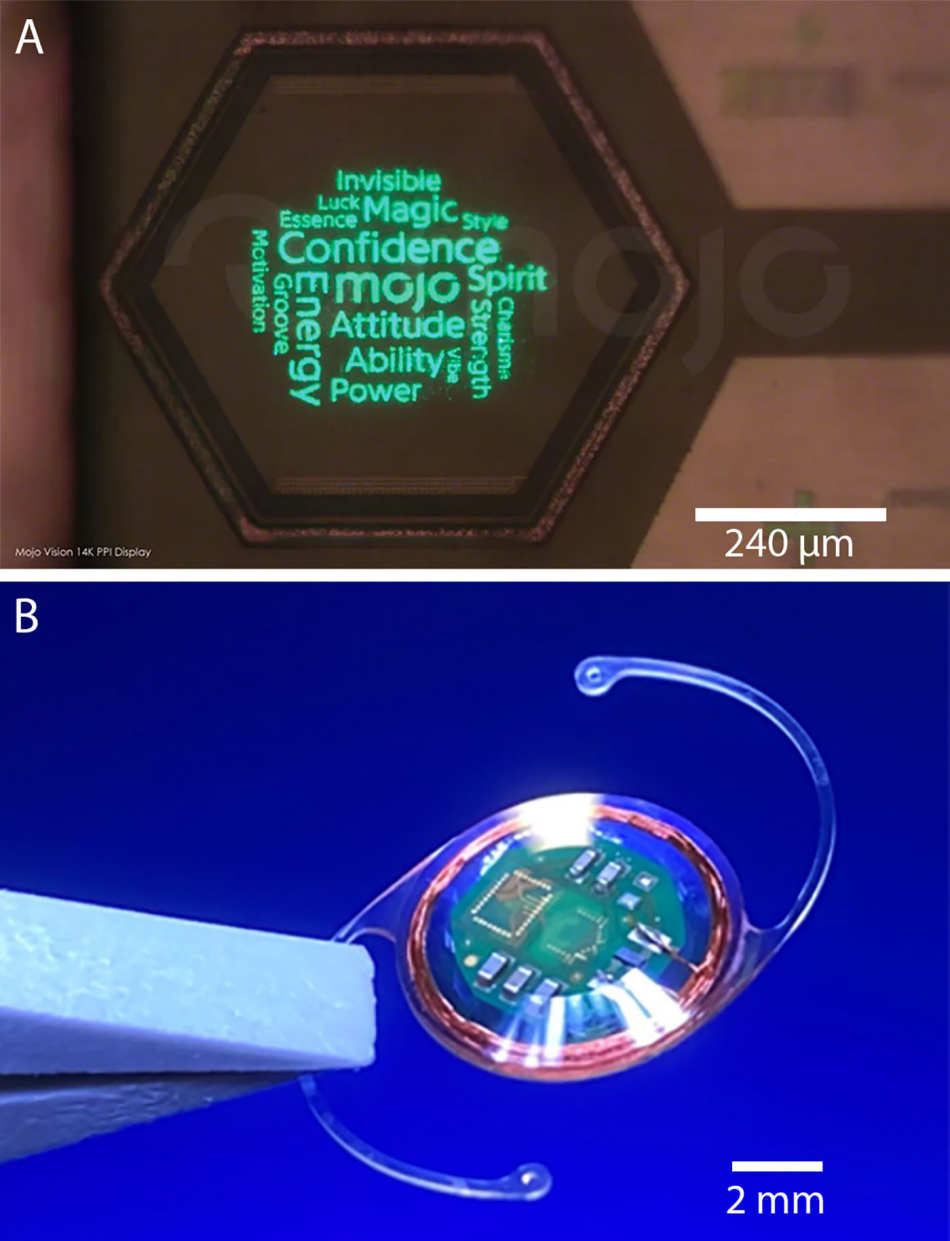
(**A**) This 14,000 pixels per inch microLED display measures only 0.48 mm across. (**B**) If implanted within the eye with supporting wireless reception electronics, this display could deliver high-quality vision to patients even in the setting of complete corneal opacity, without the need for a cornea transplant. Device produced by Mojo Vision.

**Figure 2.**
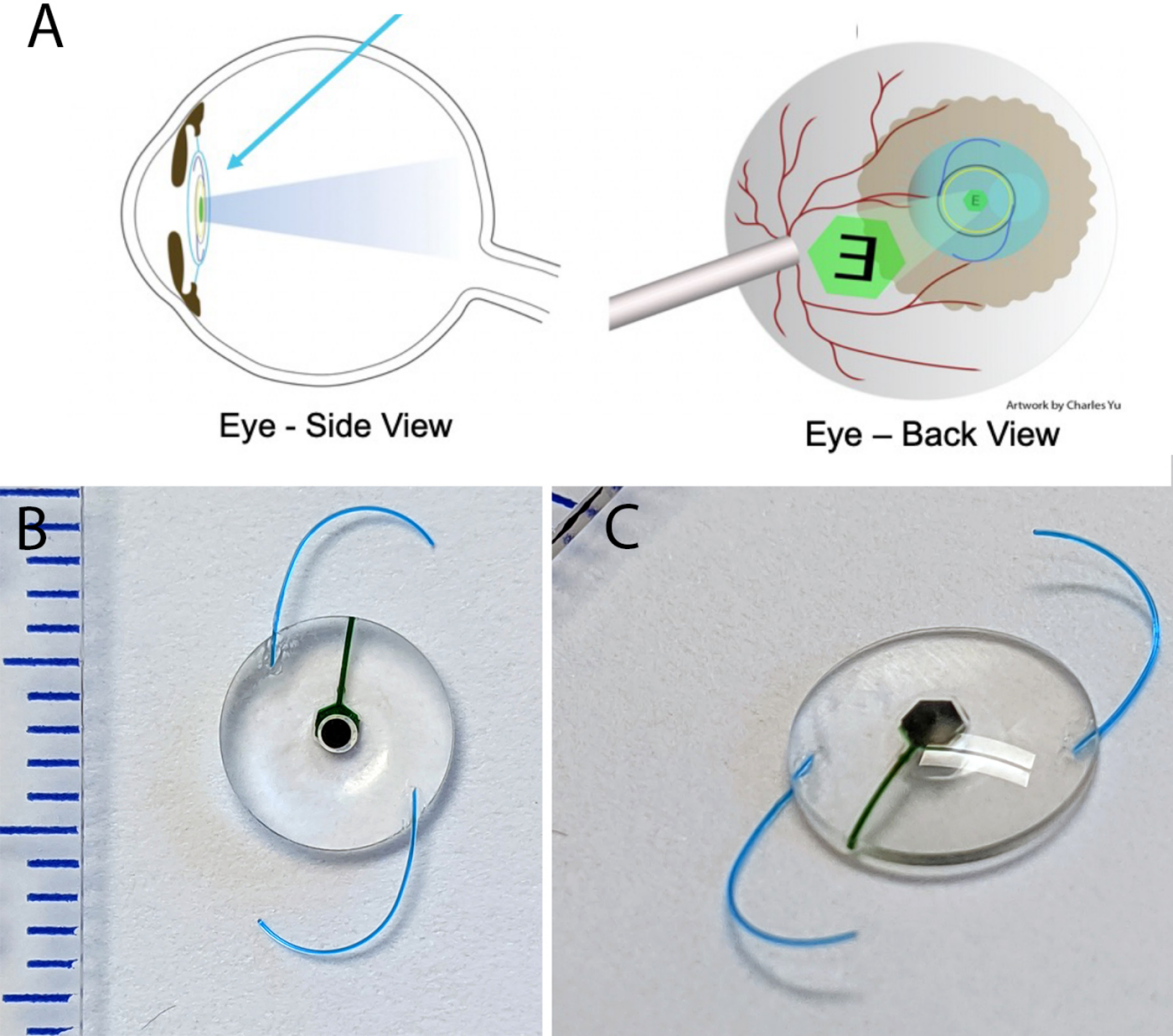
(**A**) Concept of projecting an electronic intraocular lens implanted into the lens capsule after cataract removal, placing imagery onto the retina. (**B**) Dummy implant, retina-facing side, with optical system over microdisplay. (**C**) Dummy implant, cornea-facing side.

Though all current treatments of corneal blindness seek to restore clarity to the cornea, the fundamental problem in corneal blindness is not the loss of transparency but the inability of visual imagery to reach the retina. Because the retina is normal in most patients with corneal blindness,^11^ high-quality vision is possible even in the presence of corneal opacity by placing the image source within the eye, which has become possible as electronic displays have become smaller. Previously, one study successfully reported an implant of a single LED device in rabbits, demonstrating its safety for over a year. However, this implant could only provide rudimentary light perception, far from restoring functional vision.^12^ We demonstrated high-resolution projection with a microdisplay implant measuring 5 × 7 × 7 mm, but these implants are large, difficult to implant, and are unlikely to find mass adoption.^13^^,^^14^ As microelectronic technology has improved, we have reached a stage where all needed electronics for the projection of vision inside the eye can be fit within an intraocular lens form factor. We are working with Mojo Vision (Saratoga, CA, USA) to build such a microdisplay intraocular lens. It consists of a 0.48-mm diameter microdisplay with projection optics (Fig. 1A), wireless power and data receiver, and associated electronics, placed within an IOL-shaped implant (Fig. 1B) This, when combined with an external camera and wireless video transmission system, can then be used to bypass an opaque cornea and overcome corneal blindness (Fig. 2A). Such a system can potentially deliver up to 20/20 visual acuity even with complete corneal opacity; the 14,000 pixels per inch density of the display are higher resolution than the spacing between the human fovea photoreceptors.^13^

Nonetheless, numerous variables remain to be elucidated regarding the clinical potential of this technology, the most critical of which is in vivo stability and safety of such an intraocular implant following surgical insertion. To function properly as an intraocular projection device with a fixed focal length, the implant must remain centered and stable to project focused imagery onto the macula. The study aims to provide foundational evidence supporting the viability and safety of this approach, paving the way for further development and use in humans. To provide this evidence, we conducted a 6-month safety trial of five dummy (inactive) microdisplay intraocular lens implants in rabbits.

## Methods

### Implants

Five implants were fabricated for this study by Mojo Vision. Each implant consisted of a hexagonal microdisplay, attached to a projection optic completely embedded in polymethyl methacrylate (PMMA). The central disc of the implant measured 7 mm in diameter. The central thickness was 1.3 mm, gradually tapering to 0.5 mm at the edges. The use of PMMA offers multiple advantages in this context. It is well tolerated in intraocular applications, as evidenced by its long history of use in intraocular lenses^15^; it safeguards the sensitive electronic components from the saline environment within the eye, preventing potential damage or leakage^16^; and it allows the passage of radio waves,^17^ essential for wireless data and power transmission to the implant. Specifications for the display are 0.48 mm diameter hexagonal, 256 × 256 pixels at maximum diameter, 1.8-µm pixel pitch, and monochromatic green color. The projection optic is 0.6 mm in thickness and has a magnification of 6.6×.^18^ Its focal length is designed for human eye size, not rabbit, but this does not impact the outcomes of this safety and stability study, as the primary focus is on the implant’s behavior within the eye rather than visual acuity assessments. To ensure stable positioning within the eye, two PMMA centration haptics, each 0.15 mm in diameter, were attached at the periphery of the IOL, resulting in an overall implant diameter of 13.5 mm at the apex. The implant's dry weight is 110 mg, and its submerged weight is 37 mg. These dummy devices are designed to be the same size and weight as our fully functional device but do not have functional electronics (Table 1; Fig. 2B).

**Table 1.** Specifications of Device

Specifications	Value
Display size	0.48 mm
Pixels	256 × 256
Pixel pitch	1.8 µm
Color	Green
Optic thickness	0.6 mm
Optic magnification	6.6×
Implant weight dry	110 mg
Implant weight submerged	37 mg
Implant diameter	7 mm
Implant thickness center	1.3 mm
Implant thickness edge	0.5 mm

### Animals

New Zealand White rabbits are a well-established animal model in intraocular lens research. Their widespread use is due to anatomic similarities between rabbit and human eyes, particularly in terms of lens size. This makes them suitable for studying the biocompatibility, stability, and surgical techniques related to intraocular implants.^19^ We used three female and two male animals approximately 6 months old at the time of implantation (Charles River Laboratories, Wilmington, MA, USA). This research was conducted in accordance with the ARVO Statement on the Use of Animals in Ophthalmic Research and approved by the Stanford University Administrative Panel for Laboratory Animal Care.

### Surgical Implantation

The surgical procedure for implanting the microdisplay intraocular lens followed a modified extracapsular cataract extraction technique. Rabbits were induced under general anesthesia by the veterinary team with intramuscular ketamine 25 mg/kg, midazolam 1 mg/kg, and 1% halothane inhalation. Heart rate and oxygen saturation were monitored intraoperatively. The right eye was sterilely prepped for surgery with 5% betadine. First, a 6-clock hour superior limbal peritomy was made and hemostasis achieved with bipolar cautery. Two 1-mm paracentesis incision wounds were then made to allow for the introduction of instruments and fluids into the eye. The anterior chamber of the eye was then filled with Provisc (Hilco, Mansfield, MA, USA). A ∼6-mm diameter continuous curvilinear capsulorhexis was made on the anterior lens capsule. Saline solution was injected into the lens capsule to prolapse the crystalline lens into the anterior chamber. An 8-mm shelved limbal scleral main wound was made. The crystalline lens and any residual lens cortex were then irrigated from the anterior chamber with an irrigation aspiration cannula. The now-empty lens capsule was filled again with viscoelastic. The microdisplay implant was placed through the 8-mm wound and into the capsule. The corneal incision was closed with three interrupted 10-0 Vicryl sutures (Ethicon, Raritan, NJ, USA). The conjunctiva was securely sutured with two 8-0 Vicryl sutures. No intraocular steroid or antibiotic was given.

### Postsurgical Procedures

For the first month, rabbits were given moxifloxacin 0.5% and prednisolone 1% one drop four times a day. After 1 month, no further drops were instilled. Rabbits were observed weekly for 6 months with slit-lamp examination, external photography, and tonometry (Tono-Pen; Rechert, Depew, NY, USA). At the month 1 time point, they were placed into rabbit bags, and topical tetracaine 1% was instilled in the implanted eye for A-scan biometry (Kaixin Electronic, Shenzhen, China). At the end of the 6-month trial, the rabbits were sacrificed with Euthasol (Virbac AH, Inc. Carros, France), and their eyes were fixed in formalin for histologic examination.

### Centration and Axial Stability

To determine centration stability of the implant, en face external photographs of the rabbit eyes were processed for the month 1 and month 6 time points using Adobe Illustrator (Adobe, San Jose, CA, USA). The distance of the center of the implant to the center of the cornea was quantified in the horizontal and vertical meridians. Actual distance was then calculated using these numbers. To determine the axial stability of the implant, anterior chamber depth was quantified with immersion A-scan biometry. A total of five central A-scan measurements in millimeters were taken and averaged for both the 1-month and the 6-month time points.

### Histology

At the end of the trial, the eyes were fixed in 4% formalin for 48 hours. The IOL was removed, and the eye was fixed in paraffin. It was cut into 6-µm cross sections and processed for hematoxylin and eosin (H&E) and periodic acid-schiff (PAS) staining, and ocular structures were examined under an Eclipse E800 microscope (Nikon, Tokyo, Japan).

## Results

### Surgical Implantation

**Figure 3.**
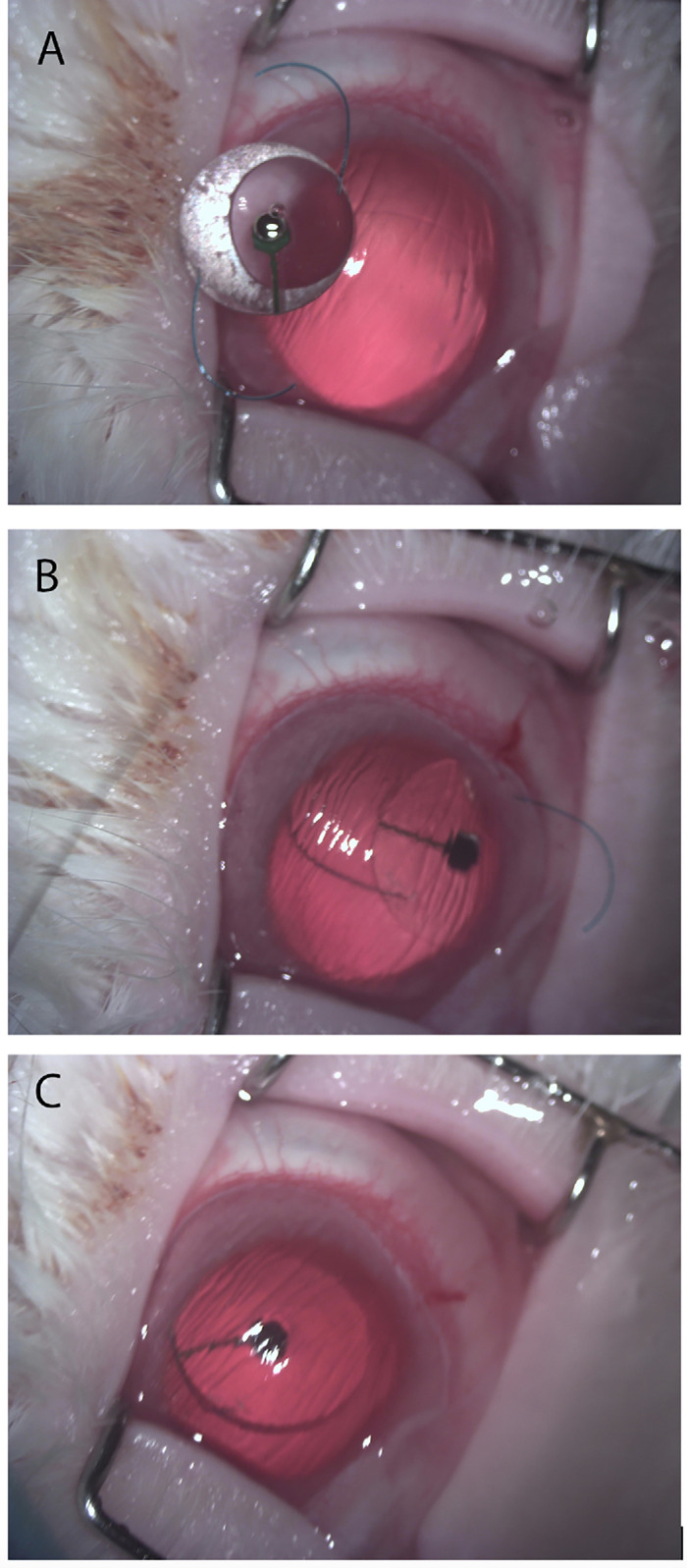
Surgical implantation technique. (**A**) Rabbit crystalline lens has been removed via a limbal incision. (**B**) The implant is placed into the lens capsule. (**C**) The limbal incision is closed.

Five surgeries were conducted as previously described without complications. All rabbits survived and recovered well. Rabbits 1, 3, and 5 had a mild pupil decentration toward the wound area immediately after the surgery, likely due to the size of the wound and tightening of corneoscleral sutures (Fig. 3).

### Postsurgical Observations and Adverse Events

**Figure 4.**
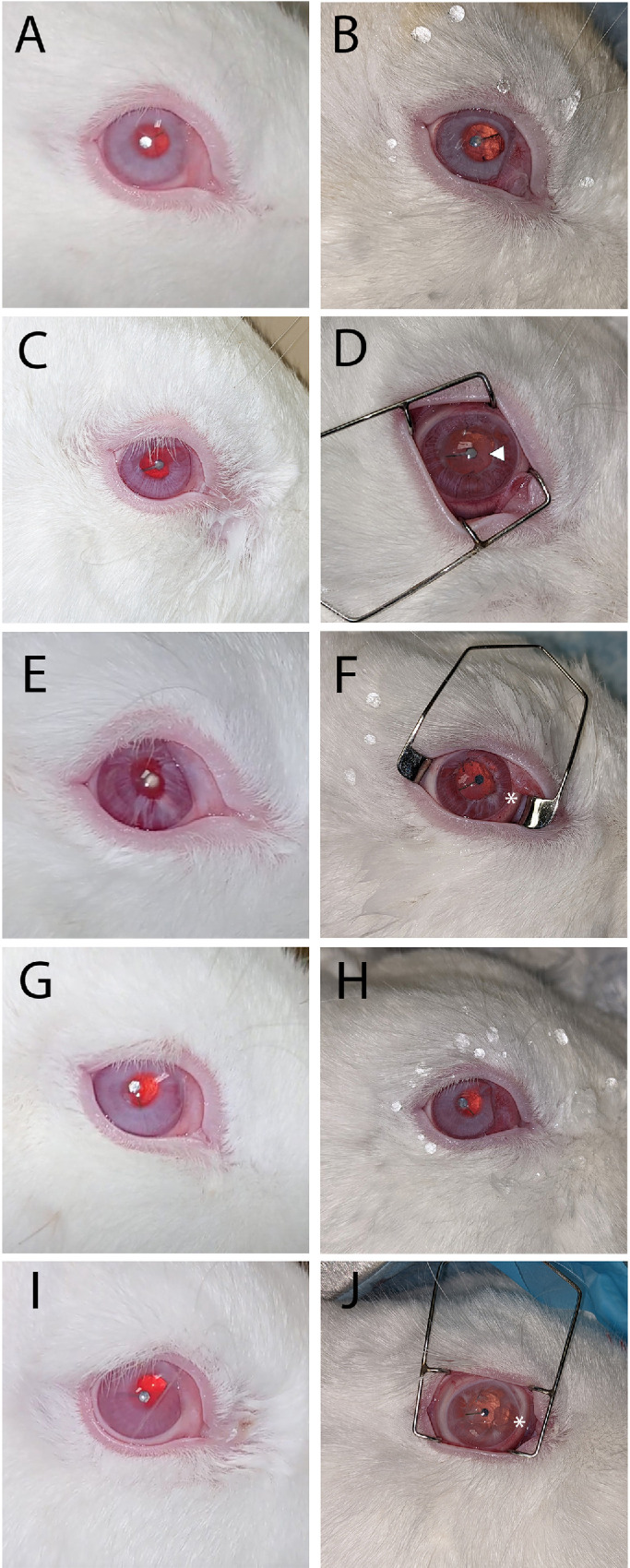
External en face photographs showing the longitudinal position of the IOL in each subject and to assess for adverse events such as PCO and synechiae. (**A**, **C**, **E**, **G**, **I**) Rabbits 1 to 5 at 1 month. (**B**, **D**, **F**, **H**, **J**) Same rabbit eyes at 6-month sacrifice. △ PCO, * iris irregularity.

All rabbits survived healthy until the end of the 6-month trial. Rabbit 1 developed mild posterior capsular opacification (PCO) at 3 months. Rabbit 2 developed iris synechiae at 1 month, mild PCO at 3 months, and moderate PCO by 6 months. Rabbit 3 developed mild irregularity postoperatively and mild PCO by 3 months. Rabbit 4 developed mild PCO at 6 months. Rabbit 5 developed iris synechia by 4 months, with iris thinning at 6 months (Fig. 4). Intraocular pressure remained below 21 mm Hg throughout all time points (Table 2).

**Table 2.** Postsurgical Observations

Subject	Observations	Mean IOP (mm Hg)
1	Mild PCO 3 months	16.8
2	Iris synechiae 1 month, mild PCO 3 months, moderate PCO 6 months	15.4
3	Iris irregularity, mild PCO 3 months	18.0
4	Mild PCO 6 months	17.4
5	Iris synechiae 4 months, iris thinning 6 months	17.7

### Centration and Axial Position

**Figure 5.**
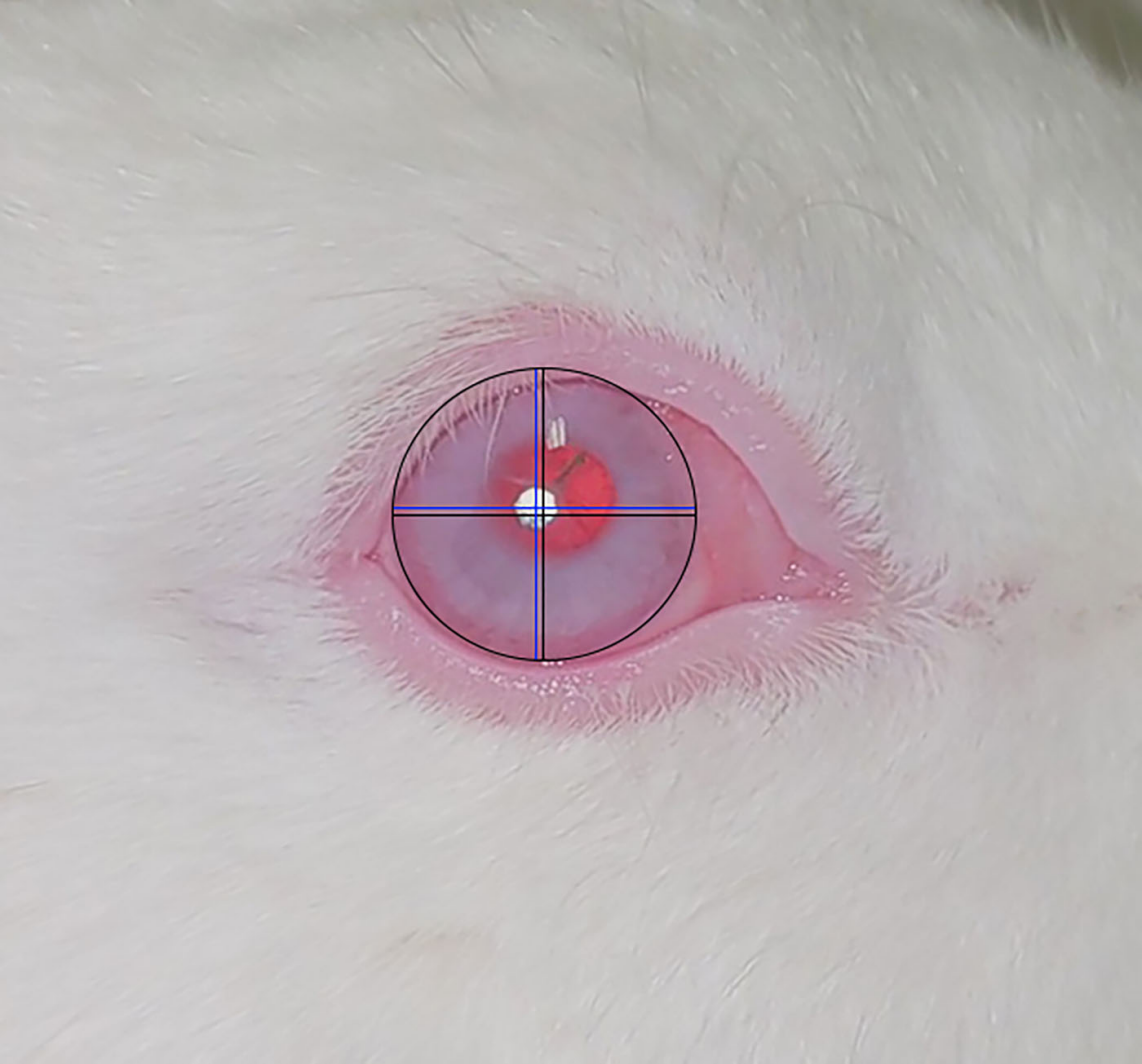
Centration was measured by the distance of the center of the implant from the center of the cornea. *Black lines* cross at the center of the cornea. *Blue lines* cross at the center of the implant.

Decentration, as measured from the center of the cornea, is shown in Table 3. Mean ± standard deviation from the midpoint of the cornea was 0.868 ± 0.442 mm at 1 month and 0.851 ± 0.591 mm at 6 months. Average change was 0.154 ± 0.124 mm (Fig. 5; Table 3).

**Table 3.** Centration and Axial Stability

	Decentration (mm)			ACD (mm)		
Subject	1 Month	6 Months	Difference	1 Month	6 Months	Difference
1	1.001	1.069	0.069	4.226	4.232	−0.006
2	0.280	0.166	−0.114	4.406	4.318	0.088
3	0.954	0.658	−0.296	4.714	4.734	−0.020
4	1.468	1.739	0.272	4.264	4.218	0.046
5	0.639	0.621	−0.018	4.198	4.252	−0.054
Mean	0.868	0.851	0.154	4.362	4.351	0.043
SD	0.442	0.591	0.124	0.213	0.218	0.032

Anterior chamber depth, used as a proxy for axial position, was measured at 1- and 6-month time points and is shown in Table 3. Anterior chamber depth was 4.362 ± 0.213 mm at 1 month and 4.351 ± 0.218 mm at 6 months. Average change in anterior chamber depth was 0.043 ± 0.032 mm (Table 3).

### Histology

Histology confirmed the presence of observed PCO. Other structures of the eye, including cornea, retina, and optic nerve, were not notably different between implanted eyes and their contralateral unimplanted eye.

## Discussion

This study presents the findings of the first animal trial investigating the long-term safety and stability of an intraocular lens incorporating an electronic projection display and optical system. This novel approach offers a potential solution for corneal blindness. In this condition, corneal opacity prevents light from reaching the retina by bypassing the cornea and projecting images directly onto the retina.

All five rabbits in the study successfully underwent surgical implantation of the device into the lens capsule, and no complications related to lens dislocation were observed throughout the 6-month follow-up period. This finding indicates that the implant’s size, weight, and material were well tolerated within the eye over an extended duration, supporting the safety and feasibility of this approach. These results were not surprising because while our device (110 mg dry, 37 mg submerged, 7-mm optic diameter) is larger than typical intraocular lenses (∼20 mg, 6-mm optic diameter), it is much smaller than the natural crystalline lens (225 mg, 10-mm diameter). In addition, we designed our implant to be below the weight of the US Food and Drug Administration–approved CentraSight intraocular telescope device (120 mg dry, 40 mg submerged).^20^ Though it was not the object of this study, we expect devices heavier than 120 mg could be implanted stably inside the lens capsule should future electronic applications require this.

There was development of at least mild PCO in all rabbits and moderate PCO in one rabbit. PCO, the growth of residual lens cells onto the capsule after cataract removal, is common with human intraocular lens implantation and even more so in rabbits.^21^ Typically, it is easily dealt with by YAG laser capsulotomy, but since we intend to use this implant in patients with corneal opacity, the issue of PCO must be carefully considered.^22^ Since YAG capsulotomy cannot be done through an opaque cornea, prophylactic posterior capsulotomy at the time of surgery may be needed to avoid a return to the operating room for surgical posterior capsulotomy. Because rabbits develop PCO more rapidly and extensively than humans,^23^ we can expect a lower rate of PCO development in humans, likely comparable to current rates with PMMA intraocular lenses. Three rabbits also experienced iris adhesions, and one had iris thinning by the end of the trial. PMMA is a material known to be well tolerated in intraocular lenses since their invention, and thus we do not think this is the cause. We believe iris contact can be avoided by altering the angle of the haptics to place the implant deeper into the lens capsule, increasing the dose of steroid eye drops, and/or pharmacologically dilating the eye. We also expect iris adhesions to be significantly less in humans than in rabbits, which are more inflammatory in general.^21^

For this visual prosthetic system to be effective, it is crucial that a clear image can be projected onto the macula. This requires stable centration as well as axial position so the image does not become excessively decentered or defocused. To address centration, we quantified the lens position at month 1 and month 6. A 0.48-mm image display with 6.6× magnification provides a projected image of 3.17 mm in diameter. The average 0.851-mm decentration would experience an approximately 25% decentered image, which can be accounted for with the calibration of the image center via software. Axial stability is important to keep the image in focus, which is even more important than centration since it cannot be corrected with software changes. We found only a 0.043-mm change in anterior chamber depth on average, which indicates a highly stable axial position. We note we only have 1-month and 6-month measurements, and longer-term studies are needed to determine stability beyond that. Our results are comparable to human IOL studies, with one study demonstrating a 0.057-mm change over 6 months.^24^

A limitation of our study related to our intended application is the use of the limbus incision extracapsular cataract extraction surgical technique in clear corneas. Because our patients will have corneal opacities, this technique is not possible, and a surgical technique involving opening the cornea will be required. However, for purposes of studying implant size, stability, and centration, it is preferable to reduce confounding factors such as large corneal incisions in the rabbits and inducing corneal opacities, which make measurement of the implant location difficult. We will conduct further trials in the future to address the exact surgical technique to be used in corneally opaque eyes. We took en face photography to measure centration, but measurements could be further improved with the use of a calibrated slit-lamp or optical coherence tomography.

Treating complicated corneal blindness with the keratoprosthesis and corneal transplantation has fundamental limitations. We seek to develop an intraocular lens electronic implant that can be used to cure corneal blindness with the excellent safety profile of cataract surgery and without any requirement for donated human tissue. An important step to validate this approach is to prove the safety of implanting such a device into the eye. In this study, we demonstrated the safety and stability of the implantation of intraocular implants with an electronic display over 6 months in five rabbits. While challenges remain in translating this technology to human patients, such as refining the surgical approach for corneally opaque eyes and addressing the long-term management of PCO, the findings of this study represent a significant step toward providing a potentially safe, curative treatment option for individuals with corneal blindness. This study provides strong evidence of the viability of our approach. The next step in our development is to complete and test powered implants. Regarding color, current microdisplays of this size allow only for one color, which is a limitation to patient function. However, it is likely that full-color microdisplays will be technologically feasible in the near future.

While this project has important implications in corneal blindness, the addition of electronic technology into intraocular lenses can have a much greater impact. With the use of larger intraocular lenses and microelectronics technology, intraocular lenses can be a platform for drug delivery, ocular and health monitoring, vision restoration, and vision augmentation. The development and implementation of electronic intraocular lens technology could mark a paradigm shift in ophthalmology, offering innovative, novel solutions for vision restoration and enhancement.
